# Association between Dietary total antioxidant capacity and knee osteoarthritis: a case-control study in the Iranian Population

**DOI:** 10.1186/s12891-024-07677-7

**Published:** 2024-07-16

**Authors:** Farshad Amirkhizi, Soudabeh Hamedi-Shahraki, Mehran Rahimlou

**Affiliations:** 1 Department of Nutrition, Faculty of Public Health, Zabol University of Medical Sciences, Zabol, Iran; 2 Department of Epidemiology and Biostatistics, Faculty of Public Health, Zabol University of Medical Sciences, Zabol, Iran; 3 Department of Nutrition, School of Public Health, Zanjan University of Medical Sciences, Zanjan, Iran; 4 Department of Nutrition, Faculty of Medicine, Zanjan University of Medical Sciences, Zanjan, Iran

**Keywords:** Knee osteoarthritis, Dietary total antioxidant capacity, Antioxidants, Case-control

## Abstract

**Aim:**

Knee osteoarthritis (KOA) is a prevalent chronic condition associated with significant pain, disability, and healthcare costs, particularly among the elderly population. Despite the considerable burden of KOA, effective treatment options for managing the condition’s underlying causes remain limited. This case-control study aims to investigate the relationship between dietary total antioxidant capacity (DTAC) and knee osteoarthritis.

**Methods:**

This case-control study was conducted on 105 patients with confirmed KOA and 210 controls. KOA was diagnosed based on the American College of Rheumatology criteria. Dietary total antioxidant capacity (DTAC) was calculated based on the ferric-reducing antioxidant power method.

**Results:**

The mean age and BMI of the participants were 53.6 ± 8.8 years old and 27.3 ± 2.7 kg/m^2^, respectively. The study participant’s DTAC score ranged from 3.56 to 25.32 with a mean and SD of 12.46 ± 5.12. In the crude model, individuals in the highest quartile of DTAC score had 71% lower odds of having knee osteoarthritis compared to those in the first quartile (OR: 0.29, 95%CI: 0.15 to 0.58, P-trend < 0.001). These associations remained significant after adjustment for potential confounders including age, sex, energy intake, family history of osteoarthritis, vitamin D and calcium use, physical activity level, cigarette smoking and BMI. Although the odds of having knee osteoarthritis decreased with increasing quartiles of DTAC in both sexes, this relationship was stronger among males than females.

**Conclusion:**

The results of this study showed that there was an inverse correlation between DTAC and KOA among the Iranian patients with KOA.

## Introduction

A chronic degenerative condition that causes pain, disability, and a poor quality of life is osteoarthritis (OA) [1, 2]. Around 240 million individuals worldwide suffer from OA, including 10% of men and 18% of women [3]. It has been reported in some previous studies that the pooled global prevalence of KOA was 16% in individuals aged 15 years old and over and 22⋅9% in individuals aged 40 years old and over [4]. There is no effective treatment for the underlying causes of KOA, even though it is pretty prevalent worldwide [5–8]. Based on the intensity, therapeutic options for KOA include medication, physiotherapy, bracing, prolotherapy, and even surgery. Non-steroid anti-inflammatory medications (NSAIDs) are the first-line treatment for KOA [9–11]. However, several studies have shown that long-term use of these drugs can have side effects such as gastrointestinal issues, increased risk of cardiovascular events such as heart attack and stroke and renal dysfunction, especially in the elderly [12–15]. Therefore, lifestyle-related approaches to prevent and moderate the severity of the symptoms of this disease have been evaluated in various studies [16–20]. One of the most critical lifestyle modification approaches is adherence to healthy dietary patterns, which are very important in preventing chronic diseases [21, 22]. Oxidative stress and inflammation have been shown to play a significant role in the etiology of KOA [17, 23, 24]. Therefore, this hypothesis has been examined in many studies to see whether dietary patterns with high levels of inflammatory foods can increase the risk of osteoarthritis and vice versa and whether foods with antioxidant and anti-inflammatory properties can reduce the risk and symptoms [25–29].

Oxidative stress is a state caused by the overproduction of reactive species, known as pro-oxidants, and the incapability of the antioxidant defense system to scavenge these species, which consequently causes irreversible impairment of joint structure and loss of articular cartilage [30, 31]. Dietary Total Antioxidant Capacity (DTAC) refers to the overall antioxidant capacity of the diet. DTAC is a measure of the collective antioxidant activity of all the different antioxidants present in the diet [32–34]. It has been reported that high DTAC can prevent the exacerbation of oxidative stress in the body and strengthen the body’s antioxidant defense system [35–37]. Therefore, the objective of the current study was to assess the association between DTAC and KOA in the Iranian population.

## Materials and methods

### Study participants

This case-control study was done from February to August 2022 on males and females aged 30 years or older. Study samples, both cases and controls, were recruited from the hospital or private clinics in Zabol County, Iran. The minimum required sample size of the study was determined based on previously published investigations that reported that higher consumption of phytochemical-rich foods decreases the chance of KOA. According to Amirkhizi et al.’s study [38], with α = 0.05, β = 0.2, the odds ratio (OR) of 0.53, and the ratio of controls to cases as 2, the sample size was determined to be 101 cases and 202 control subjects. Therefore, we recruited 105 cases and 210 controls.

Cases were individuals with new diagnosed (during the past year) of clinically and radiographically confirmed mild to moderate bilateral primary KOA. KOA was diagnosed based on the American College of Rheumatology (ACR) criteria [39] as the presence of pain in knee joint plus any three of six the following criteria: (1) age more than 50 years; (2) presence of crepitus on active motion; (3) less than 30 min of morning stiffness; (4) bony tenderness; (5) bony overgrowth; (6) no palpable warmth of synovium. The grading of KOA was done based on the Kellgren–Lawrence (K-L) grading system (grades 0–4) [40]. According to this grading system, mild and moderate KOA were considered as K-L grades of 1–2 and 3 respectively. However, the grade 4 related to the patients with severe disease, that was not included in our study.

Subjects with any evidence of secondary OA, such as those with a history of trauma, inflammatory rheumatic or septic conditions, and previous knee surgery were excluded from the study. Pregnant and lactating females and those with rheumatic diseases other than KOA and who were taking any antioxidant supplements like selenium, carotenoids, and vitamins E and C three months before enrollment on the study were also excluded. In addition, we excluded individuals with a prior history of cardiovascular diseases, endocrine disorders such as diabetes and hypo-/hyperthyroidism, cancer, renal or liver dysfunction.

Controls comprised subjects who underwent clinical and radiographic examination of the knee but were not diagnosed with KOA and were selected from the same population. Controls were frequency-matched with cases on age (± 2 years), sex, and BMI (± 1 kg/m^2^). Trained dietitians (two dietitians graduated with a master’s degree in nutrition) interviewed all subjects using a questionnaire to collect data on demographic characteristics, lifestyle habits and health-related situations.

The protocol of the research was approved by the Ethics Committee of Zabol University of Medical Sciences (Ethics No: IR.ZBMU.REC.1400.119). All participants signed voluntary written consent letter. Also, all methods were performed in accordance with the Declaration of Helsinki guidelines and regulations.

### Dietary assessment and calculation of DTAC

The usual diet information of patients was assessed through face-to-face interviews using a validated semi-quantitative food frequency questionnaire (FFQ), designed specially based on Iranian commonly consumed foods [41]. Detailed information about this questionnaire’s design, food items, and validity is described elsewhere [41, 42]. Briefly, this questionnaire records the amount and frequency of consumption of each food item during the preceding year based on a daily, weekly or monthly. The assistants helped patients estimate food quantities using calibrated household measurements (e.g., spoons, bowls, ladles). The portion size of food items eaten by each patient was converted from household measures to grams. The intake of calories and nutrient content of foods was estimated using Nutritionist IV software (First Databank; Hearst, San Bruno, CA, USA) based on the Iranian foods-modified US Department of Agriculture food composition. Nearly all foods in the participant list were coded, and non-available foods were coded to a similar item.

In the present study, DTAC was calculated based on the ferric-reducing antioxidant power (FRAP) method, which evaluates dietary antioxidants’ capability to reduce ferric to ferrous ions [43]. The DTAC power values of foods were obtained from previously published papers that provided the antioxidant capacity for each food item, determined by FRAP. The foods FRAP values are reported as mmol per 100 g of each food item (mmol/100 g) [44]. Regarding food items that DTAC data were not directly available, we considered the value of the nearest comparable food. Furthermore, if any cooked food was not directly matched with a corresponding food in a database, the DTAC value of a similar raw food was substituted. To calculate the DTAC for each patient, the consumption volume of each food item was multiplied by their related FRAP values and then summed up.

### Assessment of other variables

The measurements of weight and height were done without shoes and in light clothing for all study samples and body mass index (BMI) was calculated as weight (kg) divided by height squared (m^2^). The physical activity of participants was evaluated by the short form of the International Physical Activity Questionnaire (IPAQ), then classified into three categories of “light”, “moderate”, and “heavy” activity [45]. The IPAQ typically classifies physical activity into three categories based on the reported level of activity: light Activity, this category includes activities such as walking at a leisurely pace, light gardening, and other activities that do not significantly increase heart rate or breathing rate. Moderate Activity: Moderate-intensity activities are those that cause a noticeable increase in heart rate and breathing rate, such as brisk walking, cycling at a moderate pace, or recreational swimming. Vigorous Activity: Vigorous-intensity activities are those that substantially increase heart rate and breathing rate and require significant effort. Examples include running, fast cycling, aerobic dancing, and heavy lifting.

### Statistical analyses

Data for categorical and quantitative variables were reported as number (percentage) and mean ± SD where appropriate. Kolmogorov–Smirnov test was applied to assess the normality distribution of the data. Differences between cases and controls in demographic and anthropometric characteristics, as well as dietary intakes, were tested by the student’s *t*-test and the chi-square test for continuous and categorical data, respectively. We applied a one-way analysis of variance (ANOVA) or Mantel–Hanszel extension test to compare demographic and anthropometric characteristics of study subjects across quartiles of DTAC score, as appropriate. Significant differences in the energy,, and age-adjusted dietary intakes of participants across quartiles of DTAC score were explored using analysis of covariance (ANCOVA). The association of dietary DTAC with KOA was investigated by applying binary logistic regression in crude and multivariable-adjusted models. In the first model, age (continuous), sex (male/female), energy intake (kcal/d), family history of osteoarthritis (yes/ no), vitamin D and calcium use (yes/ no), physical activity level (light/ moderate/ heavy), and cigarette smoking (smoker/ nonsmoker) were controlled. In the second model, BMI was additionally adjusted. SPSS software version 21.0 (IBM Corp., Armonk, NY, USA) was applied to carry out statistical analyses. In the present study, *P*-values less than 0.05 were considered statistically significant.

## Results

### Characteristics of knee OA cases and controls

In this study, 315 people (105 cases and 210 controls) were recruited. The mean age and BMI of the participants was 53.6 ± 8.8 years old and 27.3 ± 2.7 kg/m^2^, respectively. Totally, 46% of study participants were male and 54% were female. The DTAC score in the study participants ranged from 3.56 to 25.32 with a mean and SD of 12.46 ± 5.12. Of the participants in the study, 77.5% (*n* = 244) were overweight or obese (BMI ≥ 25 kg/m^2^). General characteristics of KOA cases and controls are illustrated in Table 1. There was no significant difference in mean age and BMI between cases and controls. Moreover, the distributions of having normal weight, education level, marital status, family history of OA, physical activity level, and smoking condition were similar in cases and controls. However, a higher percentage of participants in the case group received more vitamin D (58.1% vs. 43.8%, *P* = 0.017) and calcium (46.7% vs. 31.4%; *P* = 0.008) supplements than the control group.

**Table 1:** General characteristics of participants by cases and controls as well as across quartiles of the dietary total antioxidant capacity ^a^

Variables		Groups		*P* ^c^	Quartiles of DTAC				*P* ^d^
		Cases (*n* = 105)	Controls (*n* = 210)		1 (*n* = 79)	2 (*n* = 79)	3 (*n* = 79)	4 (*n* = 78)	
Sex, n (%)									
Females, n (%)		56 (53.3)	114 (54.3)	0.873	38 (48.1)	44 (55.7)	45 (57.0)	43 (55.1)	0.665
Male, n (%)		49 (46.7)	96 (45.7)		41 (51.9)	35 (44.30)	34 (43.0)	35 (44.9)	
Age (years)		54.1 ± 8.5	53.4 ± 8.9	0.543	55.4 ± 9.1	53.8 ± 9.2	52.2 ± 8.7	53.1 ± 7.9	0.127
Height(cm)		163.4 ± 9.3	161.5 ± 8.6	0.73	162.5 ± 8.3	161.7 ± 7.8	163.4 ± 6.9	162.8 ± 7.2	0.66
Weight (kg)		82.3 ± 13.2	82.1 ± 11.9	0.899	81.9 ± 12.1	82.4 ± 12.7	81.3 ± 13.9	83.3 ± 10.6	0.766
BMI (kg/m^2)^		27.6 ± 2.6	27.2 ± 2.7	0.187	27.0 ± 2.7	27.2 ± 2.6	27.3 ± 2.5	27.8 ± 2.8	0.283
Healthy weight, n (%)^b^		18 (17.1)	44 (21.0)	0.423	22 (27.8)	17 (21.8)	10 (12.7)	13 (16.5)	0.088
Educational level, n (%)									
	University	14 (13.3)	32 (15.2)	0.652	6 (7.6)	18 (22.8)	12 (15.2)	10 (12.8)	0.094
	Nonuniversity	91 (86.7)	178 (84.8)		73 (92.4)	61 (71.2)	67 (84.8)	68 (87.2)	
Marital status, n (%)									
	Married	91 (86.7)	185 (88.1)	0.717	71 (89.9)	62 (78.5)	73 (92.4)	70 (89.75)	0.076
	Unmarried/divorced/widowed	14 (13.3)	25 (11.9)		8 (10.1)	17 (21.5)	6 (7.6)	8 (10.25)	
Family history of osteoarthritis, n (%)		25 (23.8)	41 (19.5)	0.378	18 (22.4)	18 (23.1)	20 (25.3)	10 (12.7)	0.207
Vitamin D supplement use, n (%)		61 (58.1)	92 (43.8)	0.017^*^	41 (51.9)	35 (44.9)	46 (58.2)	31 (39.2)	0.090
Calcium supplement use, n (%)		49 (46.7)	66 (31.4)	0.008^**^	29 (36.7)	32 (41.0)	32 (40.5)	22 (27.8)	0.285
Physical activity level									
	Light	78 (74.3)	163 (77.6)	0.535	59 (74.7)	62 (79.5)	61 (77.2)	59 (74.7)	0.910
	Moderate	23 (21.9)	36 (17.1)		16 (20.3)	12 (15.4)	16 (20.3)	15 (19.0)	
	Heavy	4 (3.8)	11 (5.2)		4 (5.1)	4 (5.1)	2 (2.5)	5 (6.3)	
Current smoker, n (%)		21 (20.0)	36 (17.1)	0.535	16 (20.3)	9 (11.5)	14 (17.7)	18 (22.8)	0.297

BMI, body mass index; DTAC, dietary total antioxidant capacity

^a^All data are reported as means ± standard deviations unless indicated.

^b^ Healthy weight was defined as BMI < 25 kg/m^2^

^c^ Determined using independent sample t-test or chi-square test, where appropriate.

^d^ Determined using the one-way ANOVA or Mantel-Hanszel extension test, where appropriate.

*p < 0.05; **p < 0.01; ***p < 0.001

The distribution of the participants in terms of anthropometric measures, socio-demographic factors, and other lifestyle habits across quartile categories of DTAC was also shown in Table 1. There were no noticeable differences across quartiles of DTAC for mean of age and anthropometric measures as well as the distribution of subjects when considering them in terms of socio-demographic factors and other lifestyle habits.

### Dietary intakes of KOA cases and controls

KOA cases reported lower intakes of dietary fiber (*P* = 0.006), vitamin A (*P* = 0.002), and vitamin C (*P* < 0.001) compared to controls. In addition, subjects with KOA consumed lower fruits (*P* < 0.001) and vegetables (*P* = 0.002) compared to controls (Table 2). Subjects in the highest quartile of DTAC score had higher energy intake (*P* < 0.001), carbohydrate (*P* = 0.002), dietary fiber (*P* < 0.001), vitamin A (*P* < 0.001), vitamin C (*P* < 0.001), refined grains (*P* = 0.002), fruits (*P* < 0.001), and vegetables (*P* < 0.001). No other noticeable difference was found in dietary intakes across quartiles of dietary DTAC score (Table 2).

**Table 2:** Dietary intakes of selected nutrients and food groups of participants by cases and controls as well as across quartiles of the dietary total antioxidant capacity ^a^

Variables			Groups		*P* ^c^	Quartiles of DTAC						*P*-trend ^d^	
			Cases (*n* = 105)	Controls (*n* = 210)		1 (*n* = 79)		2 (*n* = 79)		3 (*n* = 79)	4 (*n* = 78)		
Energy (kcal/day) ^b^			2187 ± 385	2249 ± 426	0.327	2038 ± 472		2429 ± 512		2371 ± 408	2584 ± 512	< 0.001^***^	
Nutrients													
	Carbohydrate (% of energy)		51.6 ± 12.8	54.6 ± 13.4	0.187		52.6 ± 12.2		54.4 ± 13.7	58.3 ± 15.2	59.5 ± 15.2		0.02^*^
	Protein (% of energy)		15.8 ± 4.1	14.9 ± 5.2	0.128		14.7 ± 4.2		15.1 ± 6.1	16.5 ± 5.8	15.4 ± 5.5		0.237
	Fat (% of energy)		38.4 ± 9.7	36.3 ± 8.2	0.321		37.5 ± 7.8		38.8 ± 10.1	39.1 ± 9.4	38.6 ± 9.5		0.218
	Saturated fat (g/d)		31.6 ± 11.4	30.8 ± 10.7	0.382		29.2 ± 10.3		30.4 ± 9.8	31.7 ± 11.3	31.0 ± 12.1		0.163
	Dietary fiber (g/d)		19.6 ± 3.4	24.7 ± 4.1	0.006		15.8 ± 8.9		22.5 ± 10.4	27.3 ± 12.3	30.4 ± 9.6		< 0.001^***^
	Vitamin A (RAE/d)		452 ± 287	742 ± 312	0.002		326 ± 189		419 ± 211	581 ± 270	793 ± 367		< 0.001^***^
	Vitamin C (mg/d)		108 ± 46	142 ± 61	< 0.001		89 ± 43		112 ± 77	148 ± 81	171 ± 96		< 0.001^***^
	Vitamin E (mg/d)		19.4 ± 7.3	17.9 ± 8.2	0.287		19.8 ± 7.0		20.4 ± 9.1	21.2 ± 8.7	18.4 ± 8.4		0.284
	Folate (mcg/d)		294 ± 76	321 ± 92	0.235		285 ± 82		306 ± 114	290 ± 106	311 ± 124		0.114
	Vitamin B_6_ (mg/d)		1.61 ± 0.76	2.13 ± 0.84	0. 312		1.71 ± 0.64		1.54 ± 0.80	1.94 ± 0.89	2.14 ± 1.03		0.121
	Zinc (mg/d)		11.7 ± 5.4	10.2 ± 4.8	0.106		9.1 ± 4.1		11.7 ± 6.2	10.0 ± 4.5	11.9 ± 7.1		0.233
Food groups													
	Whole grains (g/d)		105 ± 83	112 ± 91	0.458		97 ± 63		126 ± 83	110 ± 81	117 ± 94		0.293
	Refined grains (g/d)		384 ± 178	402 ± 196	0.214		318 ± 164		338 ± 188	408 ± 184	431 ± 204		0.002^**^
	Fruits (g/d)		219 ± 104	251 ± 117	< 0.001		181 ± 92		237 ± 104	279 ± 112	291 ± 127		< 0.001^***^
	Vegetables (g/d)		228 ± 92	273 ± 114	0.002		191 ± 90		216 ± 103	284 ± 131	311 ± 124		< 0.001^***^
	Legumes and nuts (g/d)		41.5 ± 23.4	44.2 ± 21.7	0.164		40.7 ± 19.4		37.5 ± 20.1	44.9 ± 21.5	41.7 ± 18.5		0.317
	Meats (g/d)		102.3 ± 51.1	92.6 ± 48.2	0.153		81.6 ± 43.1		94.4 ± 52.3	90.5 ± 49.2	87.5 ± 40.6		0.246
	Dairy products (g/d)		182.5 ± 64	191 ± 71	0.278		182 ± 45		190 ± 67	184 ± 54	187 ± 92		0.427
	Sugar-sweetened beverages (g/d)		91.8 ± 22.5	108.4 ± 26.7	0.523		81.4 ± 18.3		83.7 ± 19.6	92.1 ± 20.4	88.3 ± 20.4		0.383

DTAC, dietary total antioxidant capacity

^a^ All data is reported as means ± standard deviations.

^b^ Energy intake is adjusted for age and sex, all other values are adjusted for age, sex and energy intake.

^c^ Determined using the independent samples *t*-test

^d^ Determined using the ANCOVA

*p < 0.05; **p < 0.01; ***p < 0.001

### Association of DTAC with KOA

**Fig. 1:**
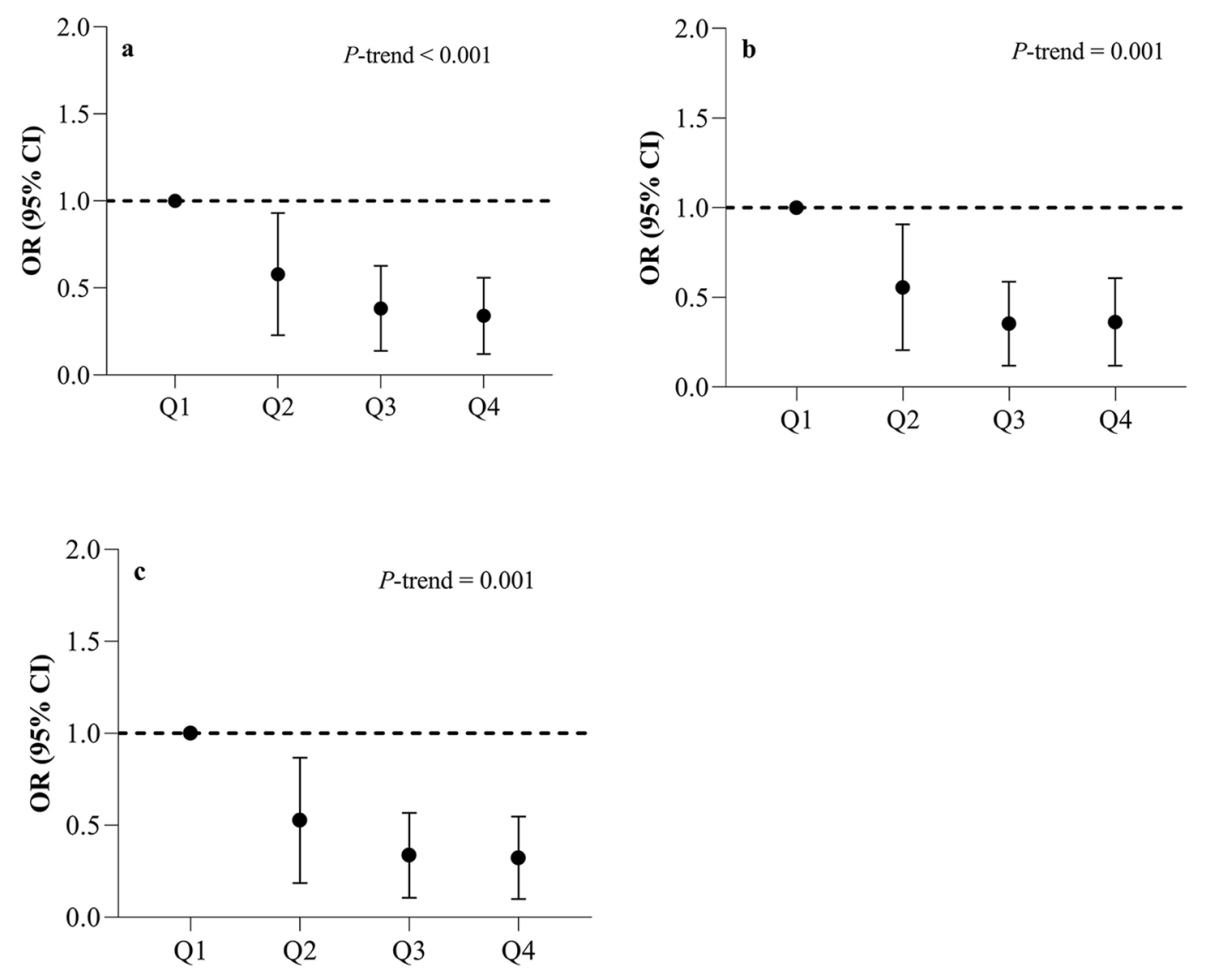
Crude and multivariable-adjusted ORs with 95% CIs for the risk of knee osteoarthritis across quartiles (Q) of dietary total antioxidant capacity. (**a**) Crude model; (**b**) Adjusted for age (continuous), energy intake (kcal/d), family history of osteoarthritis (yes/ no), vitamin D and calcium supplements use (yes/ no), physical activity level (light/ moderate/ heavy), and cigarette smoking (smoker/ nonsmoker); (**c**) Additionally adjusted for BMI (continuous). *P*-trend values were determined by the Mantel-Hanszel extension χ^2^ test

Crude and multivariable-adjusted ORs with 95% CIs for KOA are indicated in Fig. 1. In the crude model, individuals in the highest quartile of DTAC had 71% lower odds of having KOA compared to those in the first quartile (OR: 0.29, 95%CI: 0.15 to 0.58, *P*-trend < 0.001). These associations remained significant after adjustment for potential confounders including age, energy intake, family history of osteoarthritis, vitamin D and calcium use, physical activity level, and cigarette smoking (OR: 0.31, 95%CI: 0.15 to 0.63, *P*-trend = 0.001) (Model 1). After further adjustment for BMI, this inverse association between DTAC and odds of having KOA did not change (OR: 0.27, 95%CI: 0.13 to 0.57, *P*-trend = 0.001) (Model 2).

Although the interaction of DTAC and sex was not significant, however; we done a stratified analysis by sex (Table 3). Although the odds of having KOA decreased with increasing quartiles of DTAC in both sexes, this relationship was stronger among males than females. In the crude model, males (OR: 0.27, 95%CI: 0.09 to 0.81, P-trend = 0.002) and females (OR: 0.32, 95%CI: 0.13 to 0.81, P-trend = 0.013) in the top quartile of DTAC had 73% and 68% lower odds of having KOA compared to those in the first quartile, respectively. After adjustment for potential confounders (Model 1), this inverse relationship remains significant in both sexes. Further adjustment for BMI (Model 2) did not change the inverse association between DTAC and chance of knee OA neither in males nor in females.

**Table 3:** Crude and multivariable-adjusted odds ratios and 95% CIs for knee osteoarthritis across the quartiles of the dietary total antioxidant capacity, stratified by sex

Variables	Quartiles of DTAC				*P*-trend ^a^
	1 (*n* = 79)	2 (*n* = 79)	3 (*n* = 79)	4 (*n* = 78)	
Males					
NO. of cases/controls (49/96)	18/19	16/20	8/28	7/29	
Cut points of dietary TAC	< 8.11	8.11–11.48	11.49–16.05	> 16.05	
Crude	1.0	0.84 (0.34-2/12)	0.30 (0.11–0.83)	0.27 (0.09–0.81)	0.002^**^
Model 1	1.0	0.74 (0.28–1.98)	0.25 (0.09–0.75)	0.27 (0.09–0.75)	0.022^*^
Model 2	1.0	0.75 (0.29–2.03)	0.24 (0.08–0.73)	0.25 (0.09–0.82)	0.017^*^
Females					
NO. of cases/controls (56/114)	22/20	12/31	11/32	11/31	
Cut points of dietary TAC	< 8.68	8.68–12.35	12.36–15.97	> 15.97	
Crude	1.0	0.35 (0.14–0.87)	0.31 (0.12–0.78)	0.32 (0.13–0.81)	0.013^*^
Model 1	1.0	0.32 (0.12–0.86)	0.28 (0.12–0.71)	0.29 (0.11–0.75)	0.012^*^
Model 2	1.0	0.30 (0.11–0.71)	0.26 (0.11–0.72)	0.27 (0.11–0.72)	0.009^**^

DTAC, dietary total antioxidant capacity

^a^ Determined using the Mantel–Hanszel extension χ^2^ test

Model 1: Adjusted for age (continuous), energy intake (kcal/d), family history of osteoarthritis (yes/ no), vitamin D and calcium use (yes/ no), physical activity level (light/ moderate/ heavy), and cigarette smoking (smoker/ nonsmoker)

Model 2: Additionally adjusted for BMI (continuous)

*P*-trend was determined using quartiles of the dietary TAC as an ordinal variable in the models.

## Discussion

In this study, we compared participants diagnosed with KOA to those without KOA, providing insights into the relationship between DTAC and KOA among individuals in the Iranian population. The results of the present study showed that participants with higher DTAC had lower odds for KOA. Also, we found that this inverse correlation was stronger in male participants than in females.

In recent decades, a large number of studies on the consequences of increasing knee osteoarthritis prevalence have been published [46, 47]. Most existing studies have focused on the noticeable adverse of this disease’s impact on health-related quality of life that causes a substantial burden on individuals, health care systems, and the social economy [48]. Several studies have investigated the relationship between various dietary patterns and nutrient intakes with KOA. Xu et al. [49]. , in a population-based study among 2757 participants with existing KOA, examined the association between dietary pattern and progression of KOA. They found that adherence to western dietary patterns was significantly associated with an increased risk of KOA. However, subjects with prudent dietary pattern had lower odds for KOA. Also, it has been reported in a meta-analysis study that good adherence to the vegetarian diet, prudent diet and dairy intake was associated with a lower risk of KOA [50]. Vegetables, fruits and some other food groups contain high amounts of antioxidants. Therefore, diets with high antioxidant power can be a suitable nutritional approach to reducing the risk of osteoarthritis [51]. In the present study, we found an inverse correlation between DTAC and odds for KOA. In line with our findings, it has been reported that a healthy diet and Mediterranean diet, which contains higher amounts of dietary antioxidants from some dietary sources, especially fruits and vegetables, whole grains, olive oil and nuts, can reduce the risk of arthritis [52–54].

Interestingly, our study found a stronger inverse association between DTAC and KOA among males compared to females. The reasons for this sex-specific difference are not fully elucidated, but several factors may contribute to this observation.

Firstly, differences in disease prevalence and hormonal factors might play a role. According to the Centers for Disease Control and Prevention (CDC), the prevalence of KOA is higher in women than in men [55]. This higher prevalence might influence the observed associations in our study. Hormonal differences, particularly the protective effects of estrogen, may also be significant. Estrogen is thought to have protective effects against KOA, potentially mitigating the impact of dietary antioxidants in females compared to males [56]. Studies have shown that the serum estrogen levels of patients with KOA are significantly lower than those without KOA [57]. Gao et al. investigated the serum levels of estradiol and estrogen metabolites in women with and without KOA. They found that postmenopausal women with KOA had significantly lower serum concentrations of both free and total estrogen [58]. Additionally, Sniekers et al. conducted a review of studies examining the impact of ovariectomy (OVX) and estrogen treatment on animal models. Their review revealed that 11 out of 14 studies reported a detrimental effect of OVX on cartilage, and 11 out of 22 studies found that estrogen treatment had a beneficial effect on cartilage, particularly in the knee joint [59]. These findings provide substantial evidence suggesting a link between cartilage degeneration and estrogen levels.

Secondly, dietary intake and lifestyle behaviors might differ between sexes, which could influence the association between DTAC and KOA. It is possible that females in our study consumed fewer antioxidant-rich foods compared to males, leading to a potentially weaker protective effect of dietary antioxidants against KOA in females. Additionally, lifestyle behaviors and dietary patterns may differ between sexes, further impacting the observed associations. Contrastingly, a study by Hamedi-Shahraki et al. (2023) found that higher antioxidant intake was associated with a reduced risk of KOA in both men and women, suggesting that the protective effect of antioxidants may not be significantly different between sexes [60]. Moreover, estrogen might help in reducing oxidative stress and inflammation, thereby offering some level of protection against KOA. This protective effect could result in a less pronounced impact of dietary antioxidants in females compared to males [61]. Other studies have shown that the prevalence of KOA is higher in women compared to men, and this difference becomes more pronounced after the age of 50. The exact reasons for this sex-specific prevalence are not fully understood but could include factors such as vitamin D deficiency [62]. However, some research, such as that by Zhao et al. (2021), suggests that factors like vitamin D deficiency and footwear choices do not significantly account for the increased prevalence of KOA in women, indicating that other unknown factors may be at play [63]. While sex is a known risk factor for developing KOA, the exact mechanisms responsible for the observed sex-specific differences in the association between DTAC and KOA remain unclear.

Several mechanisms have been suggested for antioxidant substances’ preventive and favorable effects in preventing and modulating osteoarthritis symptoms. Among the potent antioxidants that have been studied more than other compounds are vitamin C and vitamin E. The creation of the extracellular matrix and the defense against differentiation, senescence, and apoptosis of chondrocytes are two of the many functions of vitamin C. In addition, selenium might help to restore the antioxidative capacity of chondrocytes. Vitamin E may be a powerful antioxidant causes preventing reactive oxygen species from oxidizing cell membranes. Furthermore, the protective role of vitamin E against chondrocyte-derived lipid peroxidation-mediated collagen degradation, have been well documented [29, 64, 65]. However, we didn’t find any significant difference between the case and control and between DTAC quartiles in dietary intake of vitamin E. On the other hand, we observed that people with KOA consumed significantly lower amounts of fiber compared to the control group. This is predictable because diets with a high DTAC require higher consumption of fruits, vegetables, and whole grains, which also increase fiber intake. In line with our findings, it has been reported in some previous studies that there was an inverse dose-dependent correlation between dietary fiber intake and risk of incident symptomatic KOA [66, 67]. For the positive effects of fibers in preventing the occurrence or exacerbation of KOA, several mechanisms, such as modifying the intestinal microbiome, reducing the concentration of inflammatory factors, and helping to lose weight, have been suggested [67–70].

We found that participants in the top quartile of DTAC consumed a higher amount of vitamin C and there was an inverse association between vitamin C intake and KOA incidence. In contrast to our findings, Li et al. in a cross-sectional study among 4685 participants found that there was a positive correlation between dietary vitamin C intake with the prevalence of radiographic KOA, while no significant association exists between dietary intake of carotenoid, vitamin E, and selenium, with the radiographic features of KOA. In their study, Radiographic knee OA was defined as Kellgren-Lawrence (K-L) grade 2 in at least one leg [71]. However, in another study, researchers in a clinical trial study were evaluated the effects of vitamin C supplementation among the 60 patients with KOA and the results revealed that there was an inverse correlation between vitamin C intake and KOA severity [72]. The beneficial effects of diets with high antioxidant capacity can be attributed to the presence of other compounds, including selenium, beta-carotene and vitamin A [73]. In the present study, we found that the correlation between DTAC and KOA was stronger among males than females. According to the Centers for Disease Control and Prevention (CDC) reports, the prevalence of KOA in women is higher compared to males [74]. Other studies have also shown the higher prevalence of this disease in women compared to men [74, 75]. While sex is known as a risk factor for developing KOA, the exact mechanisms responsible for this association remain unclear. It is important to clarify whether higher prevalence of KOA in females compared to males is associated with lower DTAC.

The rate of reported cases of osteoarthritis starts to differ between males and females about the age of 50 years. There is a dramatic rise in the prevalence of osteoarthritis among females starting at approximately 50 years of age, and this identified phenomenon is not seen in men [76, 77]. The exact reason for this difference has not yet been determined. However, hypotheses such as the effect of vitamin D deficiency, protective effects of estrogen, the use of high-heeled shoes, and higher obesity rates among women have been proposed to explain this difference [78–80].

The present case-control study had some limitations. First, we used the self-reported dietary assessment questionnaire, which increased the risk of dietary reporting bias. The second point is that the study’s observational nature made it impossible to investigate the causal relationship. Third, measuring the serum concentration of some nutrients, such as vitamin C and endogenous antioxidant indices, could increase the accuracy of the obtained results. Fourth, we did not include patients with severe KOA in this study because they were using different types of medications that could act as a strong confounder on our results. Finally, patients usually change their lifestyles when informed about their diseases. To minimize this error, we recruited newly diagnosed patients.

## Conclusion

In conclusion, our study findings suggest an inverse correlation between DTAC and KOA, particularly among older individuals in the Iranian population. These results underscore the potential importance of dietary interventions in KOA prevention and management, highlighting the significance of incorporating antioxidant-rich foods into the diets of individuals at risk for or diagnosed with KOA.

## Data Availability

“The data that support the findings of this study are available if anyone wants. The name and contact details of responsible person for data sharing : Mehran Rahimlou, Email address: Rahimlum@gmail.com.”
